# Mid-term survival and complications of double-crown-retained removable dental prostheses placed in the dental practice – a retrospective study

**DOI:** 10.1007/s00784-024-06090-7

**Published:** 2024-12-21

**Authors:** Anna-Luisa Klotz, Stefanie Hagspiel, Christopher Büsch, Stephanie Zenthöfer, Peter Rammelsberg, Andreas Zenthöfer

**Affiliations:** 1 Dental School, Department of Prosthodontics, University of Heidelberg, Im Neuenheimer Feld 400, 69120 Heidelberg, Germany; 2 Institute of Medical Biometry, University of Heidelberg, Im Neuenheimer Feld 130.3, 69120 Heidelberg, Germany; 3 Praxis Für Zahnmedizin Dr. Zenthöfer, Hauptstrasse 13, 69434 Hirschhorn, Germany

**Keywords:** Double crown, Survival, Complications, Prostheses, General practice

## Abstract

**Objectives:**

Clinical data on the performance of double-crown-retained removable dental prostheses (dcRDPs), especially beyond university studies, are rare**.** The objective of this retrospective clinical study was to evaluate the survival and complication-freeness probabilities of dcRDPs and to identify risk factors for failure and complications in a dental practice setting.

**Materials and methods:**

Anonymized practice data of 174 patients (56.9% female) fitted with a total of 213 dcRDPs (mean of 3.3 abutment teeth per dcRDP) were evaluated up to 11 years after prothesis placement (mean observation time of 3.9 years). Probabilities of survival and complication freeness were calculated on the prosthesis- and abutment level and visualized using the Kaplan–Meier method. Factors influencing failure and complications were computed using Cox regression analyses (R Ver. 7; alpha < 0.05).

**Results:**

During our observation period, 39 (18%) of the dcRDPs failed. The mean (95% CI) overall survival was 94% (90–97%) after 2 years and 86% (80–92%) after 5 years. DcRDP survival correlated with lower patient age and more abutment teeth (*P* < 0.05), while other analyzed factors did not. The probability of absence of prosthesis-related complications was 92% after 2 years and 80% after 5 years. The probability of no technical complications of abutments (teeth/implants) was 85% after 2 years and 78% after 5 years, and the probability of no biological complications was 87% after 2 years and 72% after 5 years. In total, 141 dcRDPs (66.2%) faced at least one complication during the observation period.

**Conclusions:**

Double-crown-retained prostheses fitted in a dental practice had acceptable mid-term survival rates and common technical and biological complications that were frequently easily manageable. However, prosthesis performance depended on the quality and number of abutment teeth/implants. Within the limitations of this retrospective analysis, the outcomes we observed in a dental practice are comparable to, albeit slightly worse than, those found in university studies.

Clinical relevance.

Evaluation of the clinical performance of dcRDPs fitted in a dental practice is important to estimate durability and complication patterns in order to weigh treatment decisions.

## Introduction

Current oral health care strategies allow many elderly people to preserve their natural teeth. However, partially edentulous spaces are frequent and need prosthetic management to rehabilitate chewing function, maintain esthetics, and preserve oral health-related quality of life [1]. For patients with severely reduced dentition, removable dental prostheses (RDPs) retained by double crowns (dcRDPs) are well-documented, satisfactory treatment options [2–4].

DcRDPs can be subdivided into three main categories based on their retention mechanism. The first is the parallel-walled telescopic crown [5] (rigid type double crown); the second is the conical crown [6] (rigid type double crown); and the third is the telescopic crown with clearance fit, coverdenture prostheses [7, 8] (non-rigid type double crown). In addition, there is a specific subtype of telescopic crown-retained RDPs that use a galvanic mesostructure made from gold. These crowns are frequently used if implants are used as dcRDP abutments [9], and allow a passive fit by intraoral luting of the mesostructure into the dcRDP framework [9, 10].

Regardless of the design of the double crown, dcRDPs provide adequate retention, good positional stability, and favorable esthetics (no clamps) of the prosthesis. They also transmit occlusal forces along the axis of the abutment teeth because of the circular relationship between the secondary crown and its abutment tooth when the prosthesis is loaded within its supportive field [5, 11]. Another advantage is that oral hygiene is facilitated by good access to the abutment teeth when the prosthesis is removed. Disadvantages of dcRDPs include the need for extensive tooth preparation to avoid excessive over-contouring of the abutment teeth and the difficult and technique-sensitive fabrication process.

Acceptable survival rates have been reported for solely tooth-supported dcRDPs. A recent systematic review found cumulative survival rates of 68.9–95.1% for rigid type double crowns and 34–94% for non-rigid type double crowns 5–10 years after fitting [12]. Schwindling et al. calculated survival probabilities of 90% for telescopic-crown-retained RDPs and 78.5% for conical-crown-retained RDPs and resilient telescopic-crown-retained coverdentures 7 years after fitting [13].

Despite these acceptable survival rates, prosthetic complications are not uncommon, but are usually easy to manage. The number of supporting abutment teeth, the abutment height, and the dentition of the antagonist jaw seem to play a crucial role in the development of technical and biological complications. The most frequently described technical complications are decementation and loss of the primary crown, veneer chipping of the secondary crown, and fracturing of the acrylic prosthesis base [2, 13, 14]. Consequently, complication-free survival of dcRDPs is lower than that of fixed dental prostheses. Biological complications can also occur, including the need for endodontic treatment, tooth fracture, and tooth loss [15].

DcRDPs can be supported by strategic implants to enlarge their supportive field (combined tooth- and implant-supported) and can be solely implant-supported in cases of edentulism [16–18]. Additional implants can be placed in patients who have fewer abutment teeth in unfavorable positions to improve prosthetic support and enhance the clinical performance [6, 19–21]. High survival rates (93.3–100% after 2–5.9 years of loading) have been reported for the superstructures and implants of solely implant-supported DCRDs [15, 19, 22–24].

However, the current literature almost exclusively reflects data collected at university hospitals, and data from general dental practice are sparse. Frisch et al. found that the most frequent complications of dcRDPs fitted in private practices are chipping of the veneer, fracture of the acrylic base or acrylic teeth, and the need for relining. However, overall survival was high and comparably to university studies [25]. Evidence on the performance of different types of dcRDP fitted in the private dental practice is missing.

### Objectives

The objective of this retrospective clinical study was to evaluate the survival and complication-freeness probabilities of dcRDPs (prothesis-level) and their anchoring abutments (abutment level: technical and biological complications) and to identify possible risk factors for failure and complications in a dental practice setting.

### Study hypotheses

The null hypothesis of this study was that there are no statistical risk factors for survival and complication freeness of the investigated dcRDPs. We also hypothesized that survival probabilities would be lower than those reported in university studies.

## Material and methods

### Population and data collection

This retrospective study was conducted in accordance with the Declaration of Helsinki and was approved by the local review board of the University of Heidelberg (no. S-136/2023). Anonymized patient data from a general dental practice in the Federal State of Hesse, Germany was analyzed. The practice owner was asked to provide the data of all patients who received a dcRDP since the practice opened in 2011. Data were extracted from electronic patient records (Dampsoft; Damp 2000; Germany) and transferred to a database. To answer the research question, predefined variables were collected for the University of Heidelberg (see below observation period and variables).

### Treatment and recall procedures

**Fig. 1:**
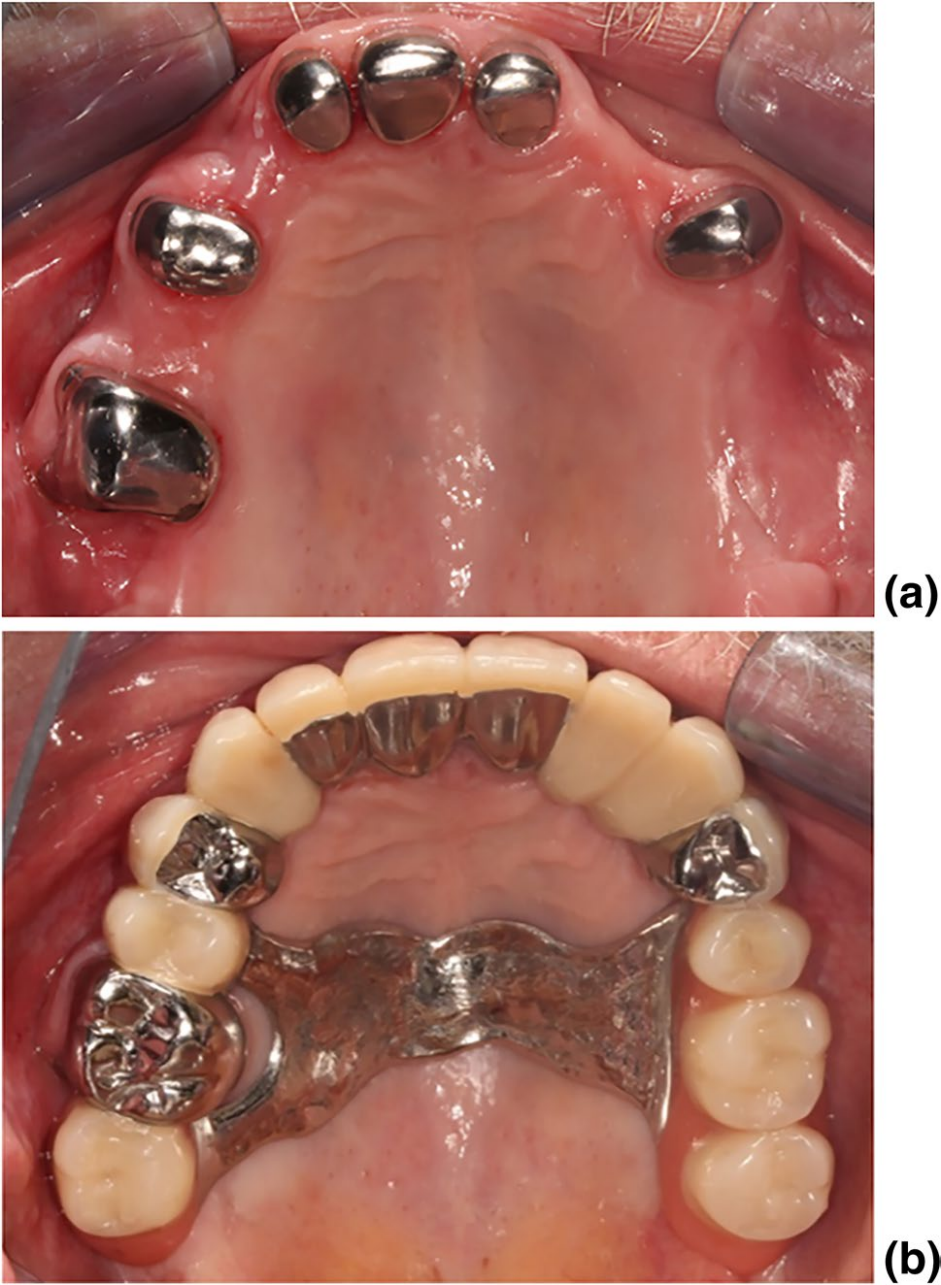
Exemplary case of a patient fitted with a conventional (tooth-supported) mandibular dcRDP, primary crowns without inserted dcRDP **(a)**, and prostheses inserted **(b)**

Patients were fitted with their dcRDPs by the owner of the practice (S.Z.) or employed dentists according to a calibrated standardized treatment protocol. Abutment teeth were prepared with a chamfer finishing line, then a conventional impression was taken using vinylpolysiloxane in dual-phase technique (Identium; Kettenbach, Eschenburg, Germany). DcRDPs were fabricated by the Flemming dental laboratory (Michelstadt, Germany) until the end of 2013, and by the Plewe Dental laboratory (Neckargemünd, Germany) from 2014 onwards. Completed primary crowns were tried in and adjusted if needed before a functional pick-up impression (Identium, Kettenbach) was taken. Next, the maxillomandibular relationship/face-bow registration was recorded, the denture wax try-in was performed, and the dcRDPs were completed. The primary crowns of dcRDPs were attached to their respective abutment teeth using a glasionomer cement (Fuji Cem, GC, Leuven, Belgium). Patients were asked to come to the practice in case of any problems and appropriate aftercare/adjustment was offered if needed. According to the quality management of the practice, all patients were scheduled for routine examinations at least once a year, but there were no fixed recall appointments as common in prospective clinical studies in the university setting. DcRDPs were tooth supported, implant supported, or tooth-implant supported and the types of dcRDPs were conventional double crown (a mixed design of telescopic and conical crowns), coverdenture, and dcRDPs with a galvanic golden mesostructure. All conventional (solely tooth supported) and coverdenture (solely tooth supported with maximal extension of the resin parts of the prostheses) dcRDPs were made from a non-precious alloy (CoCr). See Fig. 1. For galvano dcRDPs, the primary crowns and tertiary framework were made from CoCr, but the mesostructure was made of galvanic gold. These mesostructures were luted intraorally to their framework (AGC Cem Automix; Wieland Dental, Pforzheim, Germany).

### Observation period and variables

The observation period was defined as the time between placement of the dcRDP and last follow-up examination. The event-free study period was defined as the time between dcRDP placement and the occurrence of any complication or failure. Patients (called participants hereafter) were excluded from study participation if the follow-up was less than 3 months after the definitive dcRDP was placed. This allowed an adaption time of 30 days with usual refinements (such as adjustment of pressures or occlusal adjustments, which were not considered as complications) if needed and a minimum time of the dcRDPs in clinical service. Participants were also excluded from the study if the target variables to answer the predefined research question were missing from the electronic records.

**Fig. 2:**
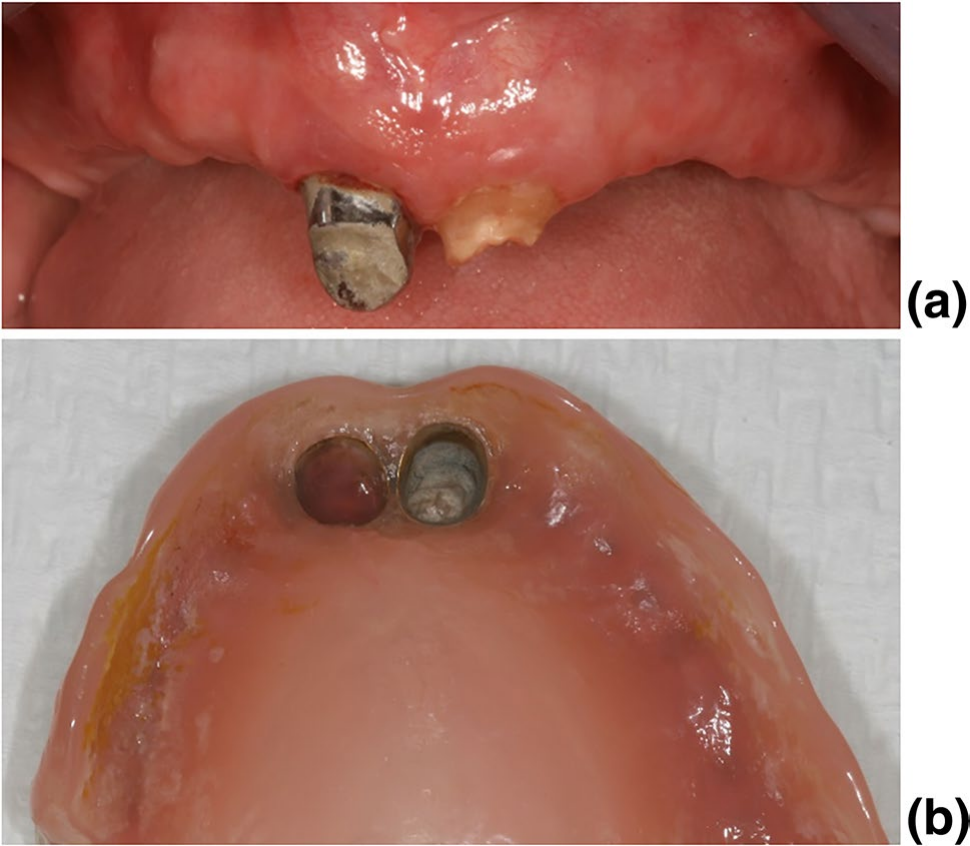
Exemplary case of a patient fitted with a coverdenture (tooth-supported) dcRDP with a complication (one primary crown is decemented, tooth 21 is fractured—> most frequent biological complication in the study), clinical situation **(a)**, corresponding dcRDP **(b)**

The following target variables were collected for each participant: age, gender, type of dcRDP, location of dcRDP, number and quality of anchoring abutments, antagonistic dentition, estimation of oral hygiene at baseline (not mandatory for inclusion), periodontal pocket depths (not mandatory for inclusion), tooth mobility (not mandatory for inclusion), vitality of the abutment teeth at baseline, failure / complications of the dcRDPs, and abutment-related biological and technical complications. See Fig. 2 for a abutment-related complication in a cover denture RDP.

### Statistical analysis

Outcomes were presented descriptively using means and standard deviations for continuous variables and absolute and relative frequencies for categorical variables. The Kaplan–Meier method was used to estimate the overall probability of dcRPD survival. Prosthesis failure was defined as the non-usability of a prosthesis due to extraction/removal of the last anchoring abutment tooth/implant (making it impossible to repair the dcRDP). In addition, this could be also due to misfit because the prosthesis was not used for a longer period of time or due to loss of the prosthesis with no possibility if the prosthesis would have been functional or not. The probability of complication freeness was evaluated on both the prosthesis level (isolated complications related to the removable parts/suprastructure of the dcRDPs, e.g. fracturing of the metal framework, acrylic base, tooth, or veneer) and the abutment level. We also distinguished between biological and technical complications. Cox proportional hazard models with mixed effects and an overall two-sided type-I error of 5% were used to isolate factors (such as age, gender, number of abutment teeth, and antagonistic dentition) that might affect the survival of dcRDPs. Abutment-related and prosthesis-related technical and biological complications were analyzed separately. The clustering of one or two dcRDPs in one participant was taken into account as a random effect in the Cox models. In the analysis of technical and biological complications on the abutment level, the covariate number of abutment teeth was removed and all possible covariates were considered. Data were analyzed using SPSS 25 (SPSS Inc. Chicago, IL USA) and SAS v9.3 (SAS Institute Inc., Cary, NC USA), and statistical significance was set at *P* < 0.05. The “coxme” package was used for Cox models and the “ggplot” package was used for data illustrations. Because all variables were included in the models, they were optimized using the Aikake approach, which also ensures that model prerequisites are not affected.

## Results

### Study population

A total of 218 patients with 269 dcRDPs were identified in patient records. Of these, 44 patients with 53 dcRDPs were excluded because the observation time was < 3 months (38 dcRDPs), the case was just a revision of an alio loco fabricated prosthesis (11 dcRDPs), there were no recalls (one dcRDP), and the dcRDPs were made again after a study prosthesis failed (three dcRDPs). In the end, 174 participants with 213 dcRDPs were included in the study. The 44 patients with 56 dcRDPs that were excluded had comparable age (mean: 69.6 years; *P* = 0.681), gender (43% men; *P* = 0.979), dcRDP location (46% maxilla*; P* = 0.254) to the included patients. However, there were significantly fewer abutments anchoring the dcRDPs in excluded participants (mean: 2.8; *P* = 0.034).

During the study period, 213 dcRDPs (649 teeth and 57 implant abutments) were placed and recalled in 174 participants. The mean (SD) age of the participants at baseline was 68.6 (12.5) years, and 92 (43%) of the dcRDPs were worn by men. The mean (SD) observation period was 3.9 (2.7) years (shortest = 3 months, longest = 11 years). Of the 213 dcRDPs included in the study, 109 (51%) were conventional dcRPDs, 82 (38.5%) were cover denture prostheses (prostheses designed in complete denture design), and 22 (10%) were dcRDPs with a galvanic mesostucture (passive fit). Of the dcRDPs, 185 were solely tooth supported, 24 were combined tooth-implant supported, and four were solely implant supported. The mean (SD) number of abutments (teeth/implants) per dcRDP was 3.3 (1.6), and 117 (55%) of the dcRDPs were located in the maxilla. All participant and prothesis characteristics are shown in Table 1.

**Table 1:** Participant and dcRDP characteristics (n = 213)

**Age**	Number of RDPs n (%)	Mean (SD)
	174 participants	968.6 (12.5)
**Gender**		
Female	121 (57%)	–
Male	92 (43%)	–
**Number of abutments per dcRDP**	–	3.3 (1.6)
**Location of dcRDP**		
Maxilla	117 (55%)	–
Mandible	96 (45%)	–
**Antagonistic dentition**		
FDP/natural teeth	54 (25%)	–
RPD	126 (59%)	–
CD	33 (15%)	–
**Type of dcRDP**		
Conventional dcRDP	109 (51%)	-
Coverdenture/resilience	82 (28%)	-
Galvanic telescope	22(10%)	-
**Support of dcRDP**		
Solely tooth supported	185 (87%)	-
Solely implant supported	4 (2%)	-
Combined tooth-implant supported	24 (11%)	-
**Abutment level (n = 706)**		
Periodontal pocket depths	n = 287	3.4 (1.4) mm
Tooth mobility	n = 683	0.1 (0.4)
Abutment region incisor	463 (65.6%)	-
Abutment region premolar	178 (25.2%)	-
Abutment region molar	65 (9.2%)	-
Root Canal Filling (yes)	91 (12.9%)	-
Oral Hygiene good	303 (54.1%)	-
Oral Hygiene improvable	257 (45.9%)	-

FDP = fixed dental prosthesis; RPD = removable partial denture; CD = complete denture; dcRDP = Double-crown-retained denture

### Prosthesis survival and probability of prosthesis- and abutment-related complications

**Fig. 3:**
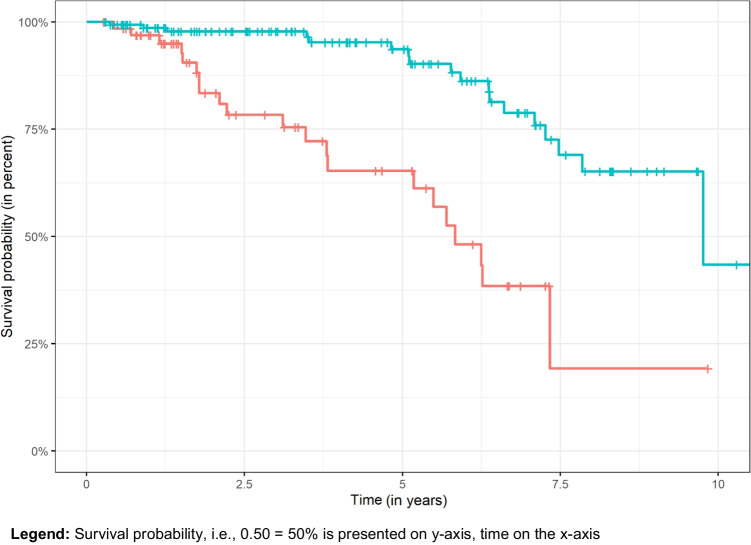
Kaplan–Meier curves for overall survival of the dcRDPs, separated for # of abutments ≥ 3 (blue), < 3 (red). **Legend:** Survival probability, i.e., 0.50 = 50% is presented on y-axis, time on the x-axis

**Fig. 4:**
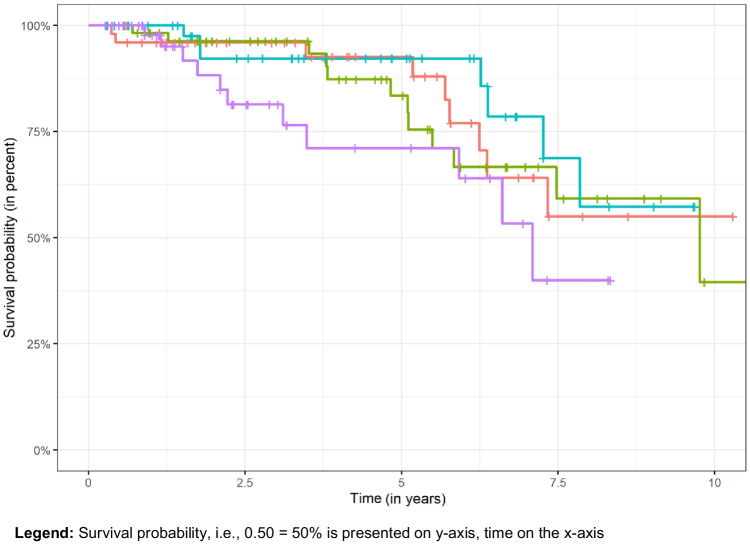
Kaplan–Meier curves for overall survival of the dcRDPs, separated for different categorized patient ages, < 61 years (red), 61–70 years (green), 71–79 years (blue) and > 79 years (pink). **Legend:** Survival probability, i. e. 0.50 = 50% is presented on y-axis, time on the x-axis

**Fig. 5:**
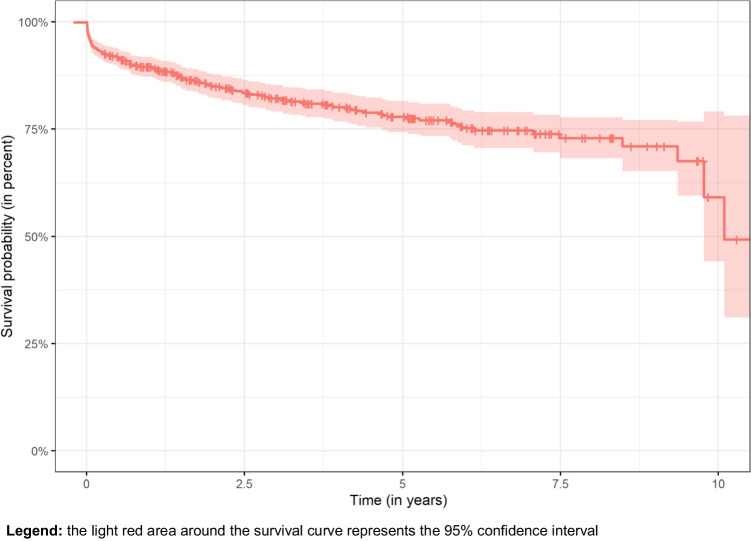
Kaplan–Meier curves for overall technical complications (abutment level). **Legend:** the light red area around the survival curve represents the 95% confidence interval

During the observation period, 39 (18%) dcRDPs failed. The overall mean (95% CI) survival probability was 94% (90–97%) after 2 years and 86% (80–92%) after 5 years. Reasons for failure were removal of last remaining abutment(s), loss or not bearing of the prosthesis, and refabrication for reasons not specified. Overall dcRDP survival (prosthesis level) correlated positively with lower patient age (*P* = 0.048) and more abutment teeth (*P* < 0.001), while no association was observed between dcRDP failure and other analyzed variables factors (gender, type of prosthesis, antagonistic dentition) (*P* > 0.05) (Table 2 and Figs. 3 and 4). Thirty-one prostheses had at least one complication (excluding abutment-related technical and biological complications) during the study period. The probability of no prosthesis-related complications was 92% after 2 years and 80% after 5 years. The total number of prosthesis-related complications was 61. The type of antagonistic dentition (RDPs) was associated with a higher risk for dcRDP complications (*P* = 0.029), while the type of antagonistic complete denture was not (*P* = 0.810) (Table 3). The probability of the absence of abutment-related technical complications was 85% after 2 years and 78% after 5 years, and the probability of the absence of biological complications was 87% after 2 years and 72% after 5 years. Almost all second dcDRPs (n = 95) had at least one technical complication of the anchoring abutments (219 abutment-related technical complications in total). These complications were more likely to occur in the mandible (HR: 1.9; *P* < 0.001) and if the abutment tooth was non-vital and had a root canal filling (HR: 3.1; *P* < 0.001), and were lower in dcRDPs with galvanic mesostructures than in conventional dcRDPs (HR: 0.2; *P* < 0.005) (Table 4, Fig. 5). The most frequent technical complications were decementation of a double crown (23%) and fracture of the core build-up-crown complex (10%). Screw loosening was observed in 9% of the implant abutments.

**Table 2:** Cox model with dichotomized overall survival of dcRDPs as dependent variable and possible independent risk factors

**95% CI**				
**Variable**	**HR**	**Lower border**	**Upper border**	***P* value**
Age	1.04	1.00	1.07	**0.048**
Gender female vs. male	0.85	0.37	1.99	0.710
Number of abutments	0.39	0.25	0.59	**0.001**
Mandible vs. maxilla	0.53	0.22	1.25	0.150
Antagonistic dentition RDP	0.33	0.11	1.00	0.052
Antagonistic dentition complete dent	0.53	0.14	2.02	0.350
Type of RDP conventional vs. coverd	1.70	0.63	4.64	0.300
Type of RDP conventional vs. galv	2.14	0.33	14.21	0.430

Significant *P* values are marked in bold. Model fit: AIC: 292; BIC: 389 (singular fit)

**Table 3:** Cox model with dichotomized complications of dcRDPs as dependent variable and possible independent risk factors (prosthesis level)

95% CI				
**Variable**	**HR**	**Lower border**	**Upper border**	***P* value**
Age	0.99	0.96	1.03	0.590
Gender	0.65	0.29	1.48	0.300
Number of abutments	0.19	0.87	1.62	0.280
Mandible vs. maxilla	0.41	0.16	1.01	0.052
Antagonistic dentition RDP	0.35	0.13	0.90	**0.029**
Antagonistic dentition complete dent	0.85	0.23	3.18	0.810
Type of RDP conventional vs. coverd	1.11	0.40	3.06	0.840
Type of RDP conventional vs. galv	0.23	0.04	1.23	0.086

Significant *P* values are marked in bold. Model fit: AIC: 291; BIC: 358 (singular fit)

**Table 4:** Cox model with dichotomized technical complications of dcRDPs as dependent variable and possible independent risk factors (abutment level)

95% CI				
**Variable**	**HR**	**Lower border**	**Upper border**	***P* value**
Age	1.01	0.99	1.02	0.470
Gender	1.12	0.75	1.66	0.590
Mandible vs. maxilla	1.92	1.31	2.83	**0.001**
Antagonistic dentition RDP	0.69	0.45	1.07	0.097
Antagonistic dentition complete dent	0.71	0.37	1.34	0.290
Type of RDP conventional vs. coverd	1.29	0.85	1.90	0.230
Type of RDP conventional vs. galv	0.15	0.05	0.44	**0.005**
Localization of abutment ant./premolar	1.02	0.67	1.55	0.930
Localization of abutment ant./molar	1.07	0.58	1.96	0.840
Implant vs. tooth	0.79	0.33	1.91	0.610
Root canal filling yes vs. no	3.10	2.02	4.75	**0.001**

Significant *P* values are marked in bold. Model fit: AIC: 1632; BIC: 1790 (singular fit)

**Fig. 6:**
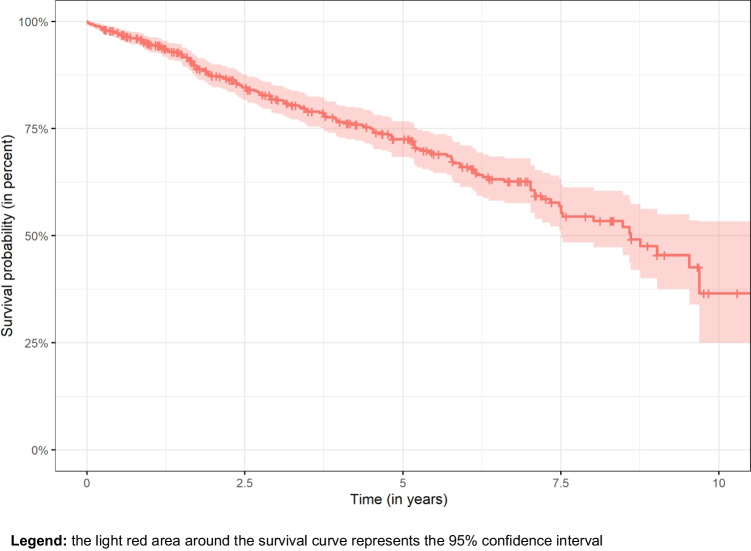
Kaplan–Meier curves for overall biological complications (abutment level). **Legend:** the light red area around the survival curve represents the 95% confidence interval

A total of 267 biological complications were observed. The most frequent biological complications in abutment teeth were tooth fracture (15%), secondary caries (4%), and need for endodontic treatment (9%). Only one implant was lost during study period. Biological complications were more frequent in cover dentures than in conventional dcRDPs (*P* = 0.007). Biological complications were less frequent in implant abutments than in tooth abutments (HR: 0.1; *P* = 0.0004) (Table 5), and the probability for survival without biological complications was 96% in implants abutments and 70% in tooth abutments (Fig. 6). Greater periodontal pocket depths at dcRDP placement (n = 287 abutments) correlated significantly with more biological complications (Pearson correlation r = 0.173; *P* = 0.003), although this was not analyzed in the multivariate models because of missing values. A significant correlation was also observed between the dichotomized estimation of oral hygiene and biological complications (n = 560; r = 0.143; *P* < 0.001). No correlation was observed between age and biological complications (*P* = 0.949).

**Table 5:** Cox model with dichotomized overall biological complications of dcRDP as dependent variable and possible independent risk factors (abutment level)

95% CI				
**Variable**	**HR**	**Lower border**	**Upper border**	***P* value**
Age	1.01	0.99	1.04	0.220
Gender	1.32	0.76	2.26	0.310
Mandible vs. maxilla	0.84	0.56	1.27	0.410
Antagonistic dentition RDP	1.18	0.65	2.14	0.570
Antagonistic dentition complete dent	2.05	0.94	4.49	0.073
Type of RDP conventional vs. coverd	2.04	0.20	3.33	**0.007**
Type of RDP conventional vs. galv	0.43	0.18	1.02	0.056
Localization of abutment ant./premolar	1.18	0.80	1.76	0.420
Localization of abutment ant./molar	1.33	0.75	2.37	0.320
Tooth vs. implant	0.11	0.03	0.37	**0.001**
Root canal filling yes vs. no	1.21	0.74	2.00	0.460

Significant *P* values are marked in bold. Model fit: AIC: 1899; BIC: 2267 (singular fit)

## Discussion

Our findings suggest that dcRDP survival and the rate of prosthesis- and abutment-related complications are satisfactory in the practice-based setting. In addition, we identified various factors that promoted survival and protected against complications. Thus, our null hypothesis had to be rejected.

Reported survival probabilities are acceptable but variable in the literature. This variance might be explained by the different types of dcRDPs with different numbers of supporting abutments, modalities, and antagonistic dentitions between clinical studies. In general, acceptable survival rates have been reported in tooth-supported dcRDPs. A recent systematic review found a cumulative survival rate of 68.9–95.1% for conventional telescopic crown-retained prostheses and 34–94% for resilient telescopic crowns 5–10 years after fitting [12]. Clinical survival rates appeared to be more favorable in implant-supported and tooth-implant-supported dcRDPs [22, 26]. In this study, we report an overall survival probability of 86% after 5 years, which falls within the survival range reported in university settings. However, our participants were fitted with three different types of dcRDPs with and without additional implant support. Previous studies have shown that abutments under resilient cover denture prostheses have a higher risk for biological complications such as tooth fracture, similar to RDPs with a more stable antagonistic dentition such as natural teeth. We did not observe the same findings in overall complication-free survival in patients fitted with a complete denture in the opposing jaw (the antagonistic dentition only affected prosthesis-related complications). One speculative reason for this could be that patients who did not wear their prostheses any longer were classified as failures. Here, one should keep in mind that our participants included individuals from a local nursing home, who may have lost or failed to wear their prostheses because of manual and cognitive impairment / dementia. However, multimorbid patient with respective impairments have also been included in studies at university hospitals. Nonetheless, our study was retrospective; participants who have lost or are not wearing their prostheses can be detected much earlier in prospective university studies with uniform recall intervals. That these cases accounted for failure, might also explain that older age comes along with a reduction of the survival probability of dcRDPs in the analysis. However, contradicting data has been presented in the literature, where a significantly higher risk of complications was found among younger and male participants [16, 13, 27]. This could be explained by the higher masticatory loads in younger male individuals [28, 29].

Our finding that implant abutment of dcRDPs reduces the number of complications is supported by the literature [26]. This is not surprising considering that this implant support improves the supportive field. In line with this, we also found that implant abutments had a higher probability of survival free of biological complications than tooth abutments after 5 years.

The estimated five-year cumulative complication-free survival for all dcRDPs was 80% in our study, with the first complication of the superstructure occurring after 3 years, regardless of severity. In the literature, complications have been reported much earlier, in the first year after prosthesis placement [30, 31]. However, it is difficult to compare the onset of complications between studies because complications are defined differently in each study. Furthermore, our Kaplan–Meier curves showed that complications in the first year are not uncommon, but they are usually easy to manage.

We observed at least one complication in 62.2% of dcRDPs during the study period, which falls within the previously published complication rates of 34.2–64.8% for dcRDPs [2, 18, 26]. The kind of complications we observed were also similar to those previously reported, including veneer chipping [16, 19, 26]. The abutment-level complications we observed were also similar to those described in previous studies, such as the loss of crown-core build-up complex in endodontically treated non-vital abutment teeth.

Although the overall survival and complication-free survival rates of dcRDPs are satisfactory, it is important to consider factors that may influence survival. The number of abutments is important for dcRDP survival [27, 32], possibly because of the more favorable (e.g., quadrangular or polygonal) arrangement, which is associated with fewer complications in tooth-supported dcRDPs [27, 32]. More abutment teeth may also promote the recovery of a dcRDP, even if single abutment teeth have to be removed.

### Strengths and weaknesses of the study

A strength of our study is that it provides rare data on dcRDP survival from a practice setting – most existing data has been generated in university settings. Another strength is the well-defined protocol and structured documentation, which makes the quality of our data comparable to that from prospective approaches. There are also limitations to our study. Our data should be interpreted and generalized with caution considering the retrospective nature of our study. Our research question was defined after the study data were gathered, which means valuable data such as periodontal pocket depths could be missing. Another limitation is that our failure rate may have been slightly overestimated because prostheses that were lost (n = 3) or did not fit any more were considered as failures. However, it was not clear whether these lost prostheses would have been and were, respectively, still functional or not. Further, valuable other information in these participants was analyzed until the failure (i.e. abutment complications). Vice versa, belated exclusion of these cases would not have clinically relevantly altered the statements of this study. Another limitation is the rather small number of patients included in the study. However, we have revealed important factors that may influence the success or failure and complications of dcRDPs. We also considered all possible variables in our models, and adjusted the best fit of the models using the Aikake approach to consider injuries of the model prerequisites and to find the best fit algorithm. In contrast to prospective studies, no fixed uniform recall appointments were available, thus different recall dates were considered using time-depending analyses. To this end, compared to other clinical studies the sample size can be considered favorable.

## Conclusion

The mid-term survival of dcRDPs placed in a dental practice showed acceptable survival rates. Observed technical and biological complications were similar to those previously described in the university setting and were easily managed. However, the outcome was dependent on the quality and the number of abutment teeth/implants. Within the limitations of this retrospective analysis, the outcomes in a dental practice appear comparable with those reported in university studies.

## Data Availability

The datasets used and/or analysed during the current study are available from the corresponding author on reasonable request.
